# Machine Learning-Assisted Automated Parametric Modeling, Simulation, and Structural Optimization of On-Chip Inductors

**DOI:** 10.3390/nano16211381

**Published:** 2026-10-28

**Authors:** Tangyi Li, Xiaonian Liu, Zhiyang Wu, Xinjie Tang

**Affiliations:** School of Physics and Electronics, Hunan Normal University, Changsha 410081, China; litangy@outlook.com (T.L.); zhiyang_wu25@163.com (Z.W.); 18508830918@163.com (X.T.)

**Keywords:** on-chip inductor, automated modeling, PSO-GA hybrid optimization, radio frequency integrated circuit

## Abstract

Aiming at the challenges of complex structural parameter spaces, excessively time-consuming full-wave electromagnetic simulations, and the tendency of traditional optimization algorithms to fall into local optima in radio frequency integrated circuit (RFIC) on-chip passive inductor design, this paper proposes an automated modeling, surrogate model, and PSO-GA hybrid optimization framework based on full-wave simulation. First, automated parametric modeling and efficient interaction with full-wave solvers are achieved through dedicated scripting interfaces. Second, a high-precision surrogate model is constructed using a physical component decoupling mechanism and Gaussian Process Regression (GPR), with the coefficient of determination (*R*^2^) for each physical component exceeding 0.970. Finally, a hybrid optimization strategy integrating Particle Swarm Optimization (PSO) and Genetic Algorithm (GA) is implemented to perform rapid inverse design under target inductance and quality factor constraints. The experimental results show that the error between the inductance and quality factor values predicted by the surrogate model and the target inductance and quality factor values is less than 1%.

## 1. Introduction

As semiconductor processes continue to evolve toward advanced process nodes (such as nanoscale FinFET and GAA structures), modern wireless communication systems have set unprecedented standards for the integration and performance metrics of radio frequency integrated circuits (RFICs). In RF front-end architectures, the performance of core modules such as low-noise amplifiers (LNA), voltage-controlled oscillators (VCO), power amplifiers (PA), and mixers depends to a large extent on the quality factor and inductance accuracy of passive components. As core passive components in RF filters, impedance matching networks, and resonant cavities, on-chip spiral inductors—whose inductance and quality factor not only directly determine the gain, noise figure, and linearity of the entire RF system but also have a decisive impact on the device’s power consumption and communication efficiency [1]. Therefore, optimizing the electromagnetic performance of on-chip inductors within a limited silicon area has become a critical challenge requiring urgent breakthroughs in the field of RFIC design.

However, as chip sizes continue to shrink, the design process for on-chip inductors faces significant physical and computational challenges. The parameter space for on-chip inductors are extremely complex, involving not only geometric variables such as line width, line spacing, number of turns, and inner diameter, but also being deeply influenced by the coupling effects of multi-layer metal interconnect structures (such as the top-layer thick metal M6 layer, bottom-layer interconnect metals, pad contacts, and bond wire effects). Traditional on-chip inductor design workflows primarily rely on full-wave electromagnetic simulation software for iterative, manual verification. Although these 3D full-wave electromagnetic simulation tools offer extremely high computational accuracy, they are fundamentally based on mesh partitioning and matrix inversion, resulting in an extremely high computational cost [2]. When dealing with multidimensional parameter spaces that require extensive exploration, simulations often take hours or even days to complete, significantly reducing efficiency.

Furthermore, there is a complex mapping relationship—characterized by high nonlinearity and multidimensional correlations—between the geometric parameters of on-chip inductors and their high-frequency electromagnetic performance metrics. In traditional design approaches, designers often rely on empirical formulas or conventional gradient descent algorithms and single heuristic optimization algorithms to find optimal solutions [3]. However, when faced with a complex nonlinear parameter mapping space, these traditional algorithms are highly prone to getting stuck in local optima, resulting in optimization outcomes that often remain in a locally suboptimal state, making it difficult to achieve a globally optimal balance between the inductor’s electromagnetic performance and its geometric structure [4].

Against this backdrop, this paper proposes a framework for the automated modeling and structural optimization of on-chip inductors. By introducing a hybrid PSO-GA, the framework replaces most of the time-consuming physical simulation iterations with machine learning surrogate models—while ensuring the target *Q*-factor and inductance values—thereby generating the optimal combination of geometric parameters for the specified *Q*-factor and inductance values [5].

The rest of this paper is divided into three sections: Section 2 describes the design logic behind the automated modeling of on-chip inductors and the PSO-GA hybrid optimization algorithm, while also providing a detailed account of the development of the machine learning surrogate model and the dataset creation process. Section 3 summarizes the simulation results, analyzes algorithm convergence, and compares the performance of the design optimization methods. Section 4 concludes the paper.

## 2. Automated Modeling and Structural Optimization Algorithms

**Figure 1:**
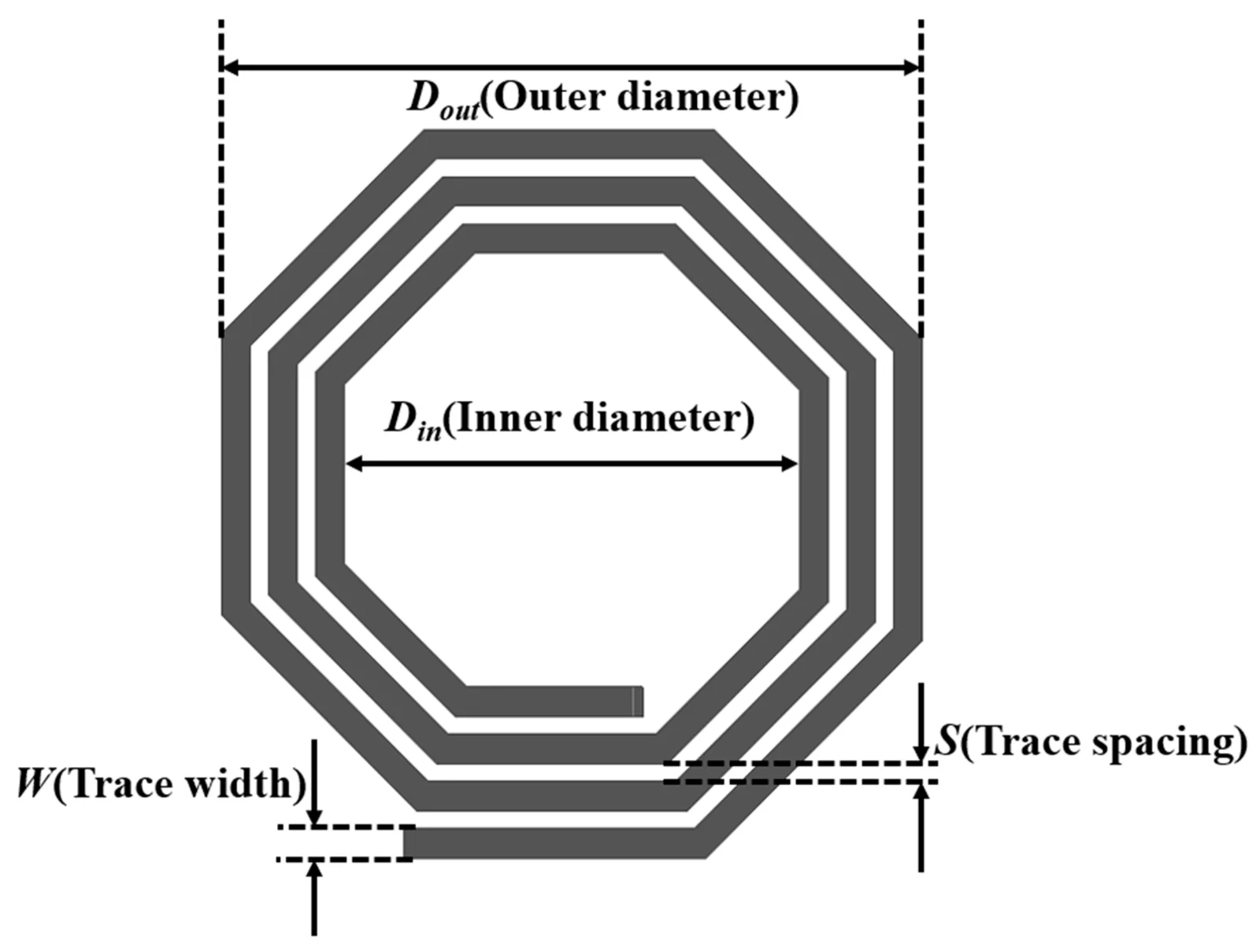
Schematic diagram of an on-chip octagonal helical inductor.

This study designs and implements a design framework consisting of automated on-chip inductor modeling and electromagnetic simulation, a PSO-GA hybrid optimization algorithm, and a Gaussian Process Regression (GPR)-based machine learning surrogate model. This framework aims to address the issues of low efficiency in manual simulation and the tendency of optimization search spaces to fall into local optima in traditional RF inductor design. The on-chip inductor structure used in this paper is shown in Figure 1.

### 2.1. Automated Modeling and Simulation of On-Chip Inductors

The automated simulation establishes direct communication with Ansys HFSS via the Python-based PyAEDT library to achieve automated modeling and simulation of on-chip inductors. During the parametric modeling stage, the script dynamically adjusts the geometric features of the on-chip octagonal helical inductor—such as line width, line spacing, number of turns, and inner diameter—according to predefined variable ranges. Subsequently, the system automatically completes mesh generation, port assignment, and solution tasks, while processing the extracted S-parameters in real time into key performance metrics such as inductance and quality factor. This process establishes a closed-loop workflow from geometric parameter input to performance metric output, with a single simulation taking only a few minutes, which dramatically reduces the data acquisition cycle. The design frequency for this article is 2.5 GHz, with a target inductance of 1.6 nH and a target quality factor of 15.5. The value ranges of the geometric parameters for the on-chip inductors are listed in Table 1.

### 2.2. Gaussian Process Regression

Gaussian Process Regression (GPR) is a machine learning method grounded in Bayesian theory and non-parametric estimation [6]. Unlike traditional deterministic regression models, GPR assumes that the function values corresponding to any finite set of input data follow a joint Gaussian distribution. Owing to its capability for probabilistic prediction (simultaneously providing the mean and predictive uncertainty/variance), along with its exceptional generalization performance and high fitting accuracy when handling small-sample, high-dimensional, and non-linear regression problems, GPR is exceptionally well-suited for constructing surrogate models for computation-intensive on-chip passive devices characterized by complex parameter relationships [7].

A Gaussian process is fully specified by a mean function and a covariance function. Given the training data, the predictive mean and variance of test points are derived through conditional Gaussian distributions, thereby yielding the predicted values concurrently. The mean function primarily provides a baseline for the samples, around which the samples are distributed. If the mean function approximates the sample data closely, the prediction uncertainty will be substantially reduced; therefore, the mean function is typically set to zero in practice. The covariance function mainly describes the correlation between input points and simultaneously acts as the kernel in Gaussian process machine learning, utilizing inter-point relationships and input samples to predict values at unknown locations [8].

### 2.3. Structural Optimization Algorithm

#### 2.3.1. Particle Swarm Optimization Algorithm

Particle Swarm Optimization (PSO) algorithm is a heuristic global optimization algorithm inspired by the collaborative behaviors of natural swarms, such as bird flocking or fish schooling. When applied to solving extreme values of complex functions, mechanical design optimization, or machine learning parameter tuning, PSO does not require the objective function to be differentiable, can effectively avoid falling into local optima, and features a simple structure, few parameters, and fast convergence [9].

The movement of a particle within the swarm is determined by its current velocity and position, individual experience (its historical best position), and global experience (the swarm’s historical best position). Assuming a swarm containing *K* particles, the position of each particle, *X_k_ = (X_k_*_1_, *X_k_*_2_…, *X_kn_)*, represents a candidate solution defined in an N-dimensional solution space [10]. During the iterative process, each particle in the swarm moves to a new position in the solution space with a velocity *V_k_ = (V_k_*_1_, *V_k_*_2_…, *V_kn_)*, updating every dimension accordingly, such that

(1) $$V_{kn}\;=\;wV_{kn}\;+\;c_{1}r_{1}\;\left(pbest_{kn}\;-X_{kn}\right)+c_{2}r_{2}\;\left(gbest_{kn}\;-X_{kn}\right)$$

where *w* is the inertia weight, used to represent the degree to which a particle maintains its previous motion trend; *c*_1_ and *c*_2_ are learning factors, which determine the extent to which the particle is influenced by its best position (also referred to as personal best or cognitive component) and the swarm’s best position (also referred to as global best or social component); and *r*_1_ and *r*_2_ are independent scalars randomly generated within the interval [0, 1], imparting random perturbations to the search process to prevent the swarm from suffering premature local stagnation [11].

#### 2.3.2. Genetic Algorithm

Genetic Algorithm (GA) is a class of stochastic global optimization algorithms simulating the biological evolution process in nature, representing an important branch of evolutionary algorithms [12]. By mimicking biological evolutionary mechanisms, a group of candidate solutions undergoes continuous selection, recombination, and mutation across iterations, ultimately retaining individuals with high fitness to approximate the optimal solution of a problem.

In genetic algorithms, a set of parameters defines an individual, and a set of individuals defines a population. Genetic algorithms typically begin by randomly generating an initial population consisting of several individuals, and then calculate the fitness of each individual to evaluate its quality; this fitness is usually the value of the objective function in the optimization problem being solved [13]. The algorithm then proceeds through iterations, with each iteration representing a generation. Based on a probabilistic selection mechanism of “survival of the fittest”, high-fitness individuals from the parent population are selected. These are then subjected to crossover, recombination, or mutation with a certain probability to form a new generation of offspring. The new generation of candidate solutions is then used in the next iteration of the algorithm. The algorithm terminates when a satisfactory level of fitness is reached or when the maximum number of iterations is reached [14].

Despite their exceptional performance in solving complex non-linear, multi-modal, and discrete optimization problems, genetic algorithms still suffer from limitations and drawbacks in practical engineering and academic applications, such as premature convergence and a tendency to fall into local optima, as well as parameter sensitivity and tuning difficulties.

### 2.4. Design of a Surrogate Model and Structural Optimization Algorithm Based on Gaussian Process Regression (GPR)

Directly mapping geometric parameters to nonlinear higher-order performance metrics (such as the quality factor *Q*) often leads to a decline in the generalization capability and extrapolation accuracy of surrogate models. To address this, this paper introduces a physical component decoupling mechanism. Using a single-π equivalent circuit model of on-chip inductors, the electromagnetic response of the inductor is decoupled into three core physical parameters: series inductance, series resistance, oxide-layer parasitic capacitance. Serving as a bridge for preliminary performance evaluation, this model establishes a nonlinear mapping relationship between geometric parameters and inductor performance (quality factor and inductance value) [15]. It can replace physical simulations that take several minutes with predictions at the millisecond level, thereby providing efficient performance feedback for subsequent optimization and search processes.

**Figure 2:**
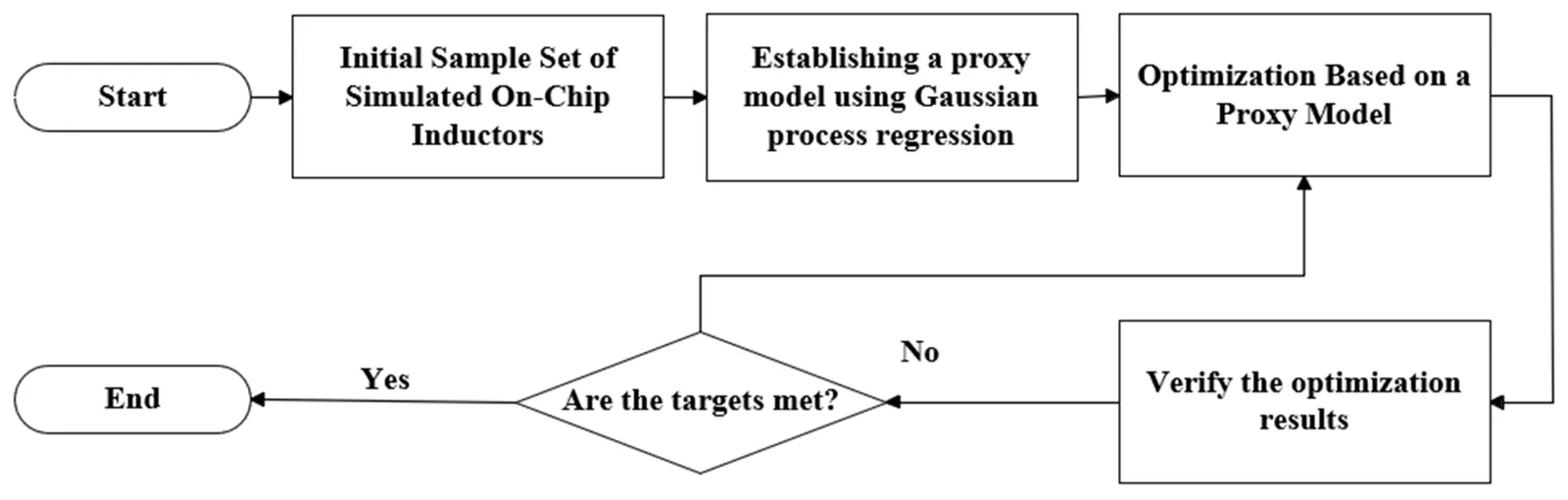
Flowchart for automated modeling and optimization of on-chip inductors.

After the surrogate model was constructed, a hybrid PSO-GA optimization algorithm was introduced to optimize the inductor’s performance. This algorithm leverages the strong global search capability of Particle Swarm Optimization (PSO) to rapidly converge within the design space and approach the region of optimal performance. A Genetic Algorithm (GA) was also incorporated; by simulating biological mechanisms of mutation, the GA introduces random perturbations to the population of candidate solutions, preventing the algorithm from getting stuck at local optima. This hybrid mechanism achieves a deep balance between the algorithm’s convergence speed and its ability to explore the search space. Combined with the rapid performance prediction provided by the GPR surrogate model, it significantly reduces the number of real-time calls to the physical simulator, thereby enabling rapid optimization of on-chip inductor performance while ensuring design accuracy [16]. The automated modeling and algorithm optimization workflow is shown in Figure 2.

## 3. Simulation Experiments and Analysis of Results

This study validated the aforementioned automated design framework. The experiments were designed to evaluate the accuracy and efficiency of the algorithm in finding the optimal geometric parameters for on-chip inductors under given constraints on the target inductance and quality factor [17]. Using the PyAEDT co-simulation platform, parameter space sampling was performed for an on-chip octagonal helical inductor, successfully constructing a dataset of 1106 valid electromagnetic simulation samples. This dataset was used to train and validate the GPR prediction models for physical components such as *L_s_*, *R_s_*, and *C_ox_*. The evaluation metrics used were the coefficient of determination (*R*^2^) and the mean absolute percentage error (MAPE) [18]. The simulation environment settings for on-chip inductors in HFSS are as follows: the substrate material is silicon, which has a relative permittivity of 11.9, a conductivity of 0, and a resistivity of infinity; the metal thickness is 4 µm; the metal lines material is copper, which has a conductivity of 58,000,000 S/m; set the outer surface of the air box as a radiative boundary; the port uses lumped port.

The experiment established specific target performance metrics, requiring the algorithm to find the optimal geometric parameters that meet these targets while satisfying the target inductance value and quality factor. The experimental results show that, through iterative optimization using the PSO-GA hybrid algorithm, this framework can identify a set of geometric parameters that limit the deviation of the resulting inductor performance from the target values to within 1%. Compared to traditional manual design, this framework not only meets the mandatory requirements for inductance and quality factor but also identifies superior geometric structures through global search [19].

### 3.1. Analysis of Prediction Accuracy and Trends in Physical Components of Surrogate Models

**Figure 3:**
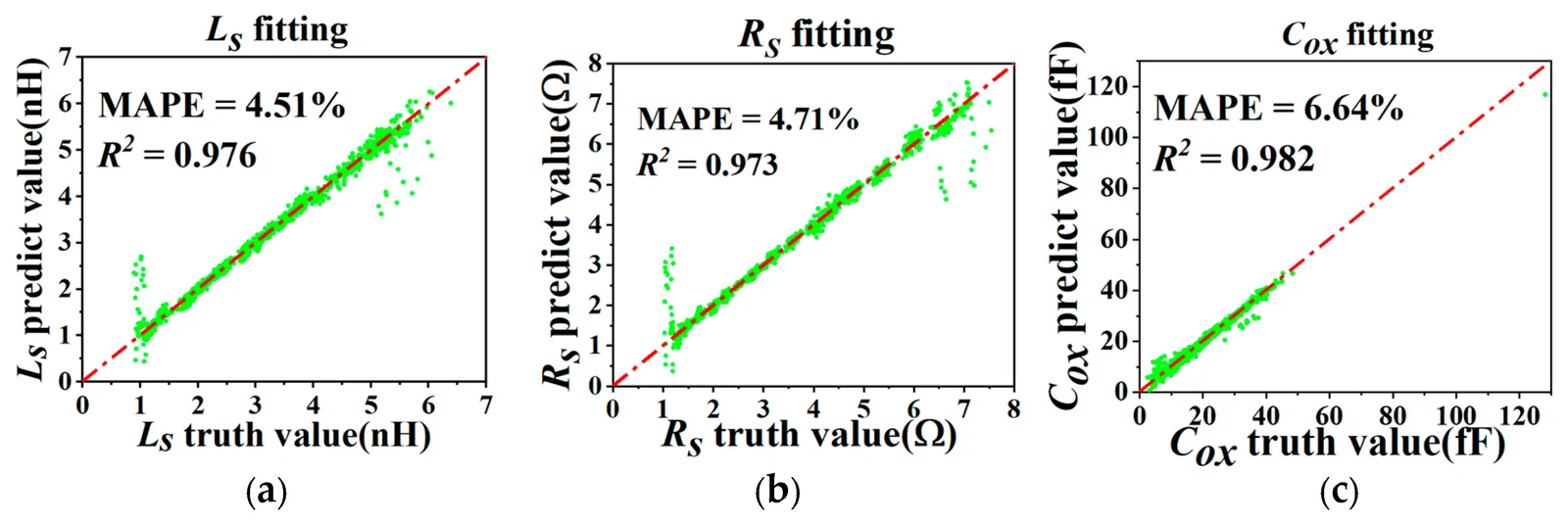
Comparison chart of key parameter fitting results for on-chip inductors based on GPR models: (**a**) Graphs showing the prediction accuracy of series inductance; (**b**) Graphs showing the prediction accuracy of series resistance; (**c**) Graphs showing the prediction accuracy of oxide layer capacitance.

To verify the reliability of the Gaussian Process Regression (GPR) surrogate model, this paper models the decoupled physical components—including *L_s_* (series inductance), *R_s_* (series resistance), and *C_ox_* (oxide layer capacitance)—separately and trains the model using a combination of the Gaussian kernel and the white noise kernel. A scatter plot of fitting accuracy was used to compare and validate the actual on-chip inductance data at the 2.5 GHz frequency with the GPR predictions. The coefficient of determination (*R*^2^) for each parameter reached 0.970 or higher, indicating that this surrogate model can accurately predict the results of full-wave electromagnetic simulations. The evaluation of the prediction accuracy of the Gaussian Process Regression surrogate model for each physical component is shown in Table 2 and Figure 3.

In addition, to further verify whether the patterns captured by the GPR surrogate model are consistent with the physical nature of micro- and nano-scale RF electromagnetic fields, this paper conducted a univariate sensitivity scan analysis. Under the reference conditions of a fixed number of inductances turns (*N* = 3), a line spacing of 4 μm, and an inner diameter of 80 μm, a univariate continuous scan was performed, varying the line width from 6 μm to 12 μm to observe the trends in the effects on the physical components of decoupling:

1. The series resistance *R_s_* exhibits a decreasing trend: as the line width increases, the cross-sectional area of the conductor increases, alleviating the skin effect and current crowding at high frequencies, and significantly reducing both DC and high-frequency ohmic losses. The GPR model accurately predicts that the series resistance *R_s_* follows a smooth, monotonically decreasing curve as the line width increases, which is fully consistent with the theory of electromagnetic losses in microwave engineering.
2. The series inductance *L_s_* exhibits nonlinear fine-tuning: as the wire width increases, although the conductor’s cross-sectional area increases, causing a slight adjustment in the magnetic flux distribution, the changes in self-inductance and mutual inductance exhibit a gradually decreasing nonlinear characteristic due to constraints on the turn spacing and overall dimensions. The GPR model accurately captures this subtle yet critical nonlinear inflection point, thereby avoiding the potential biases associated with linear models.

### 3.2. Convergence Performance of the PSO-GA and Reverse Optimization

**Figure 4:**
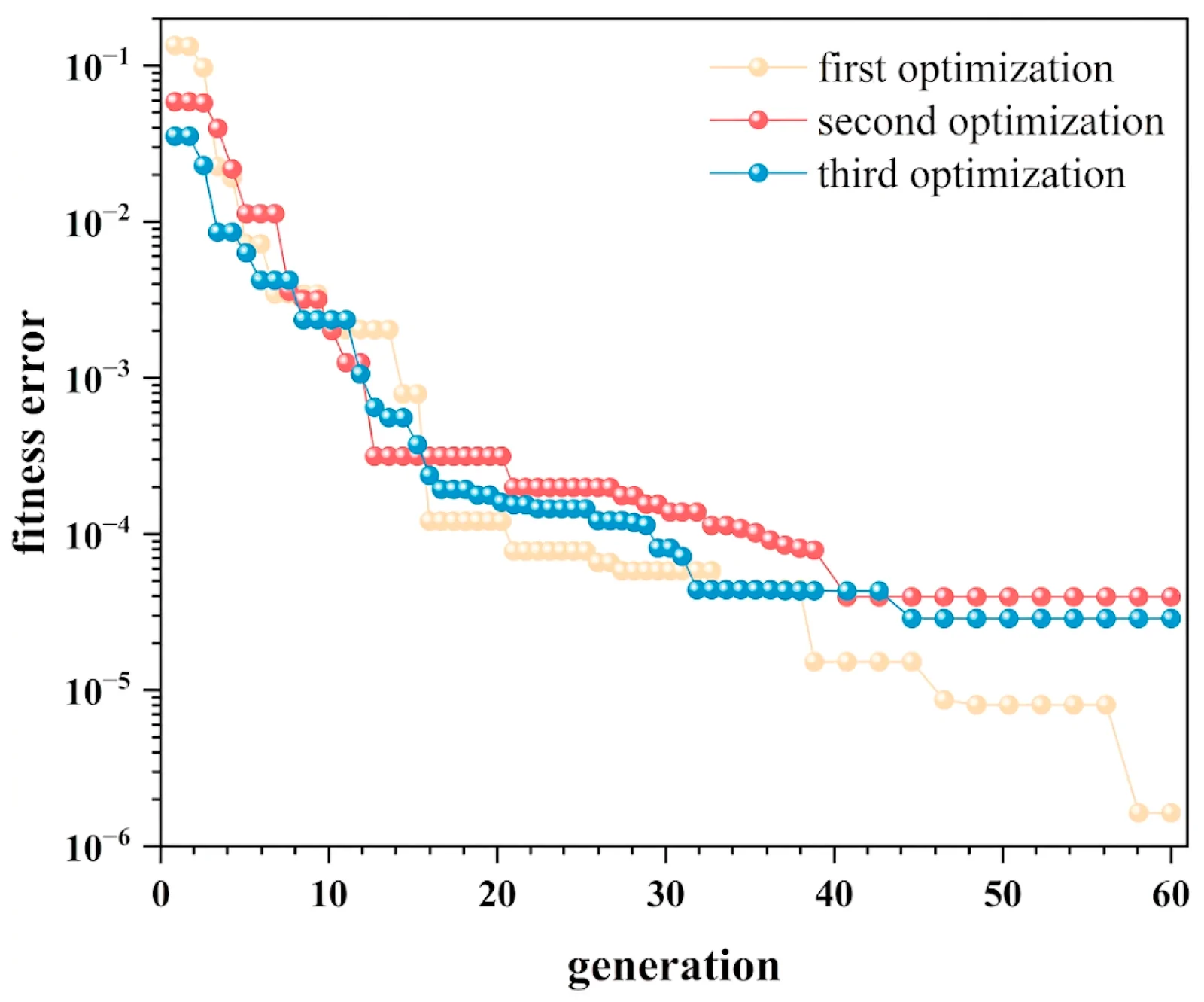
Convergence curve plot of a hybrid optimization algorithm.

The objective function for optimization uses a weighted relative error function based on the quality factor *Q* and the inductance value *L*:

(2) $$f(x)=\frac{\left|Q_{pred}(x)-Q_{target}\right|}{Q_{pred}}+\frac{\left|L_{pred}(x)-L_{target}\right|}{L_{target}}$$

where ***x*** is the combination of inductor geometry parameters, *f*(***x***) is the sum of prediction error of quality factor *Q* and inductance value *L*. The particle swarm optimization algorithm is configured with a population size of 30 and a maximum number of iterations of 60. The learning factors *c_1_* and *c_2_* are set to 1.2, the inertia weight *w* is set to 0.6, and a mutation operator with a 15% probability is introduced. The target performance metrics are set to *Q_target_* = 15.5 and *L_target_* = 1.6 nH. The algorithm convergence curve and results for the recommended parameters are shown in Figure 4, along with the accompanying notes.

The optimal combination of geometric parameters obtained is: line width *W* = 6 µm, line spacing *S* = 2 µm, number of turns *N* = 3, and inner diameter *D_in_* = 67 µm. Substituting these values into the physical model for prediction yields *Q_pred_* = 15.45 and *L_pred_* = 1.587 nH at 2.5 GHz, with a total error of less than 0.9%, satisfying the design accuracy requirement of keeping the deviation from the target values within 1%. The optimization process took only about 4 s, achieving a several-thousand-fold increase in efficiency compared to traditional Electromagnetic Simulation parameter sweep iterations.

At the same time, to evaluate the overall performance of the hybrid intelligent optimization algorithm proposed in this paper for on-chip inductor inverse design, this section presents multiple sets of comparative experiments. The optimization strategy adopted in this paper is compared side-by-side with traditional global optimization algorithms (such as the standard genetic algorithm and the standard particle swarm optimization) as well as the traditional direct brute-force method using full-wave electromagnetic simulation. Under identical target performance constraints (target quality factor *Q_target_* = 15.5, target inductance value *L_target_* = 1.6 nH) and the same hardware runtime environment, each algorithm was run independently 20 times. The actual optimization performance of each algorithm is shown in Table 3.

Through an in-depth analysis of the results of the comparative experiments described above, the following conclusions can be drawn:

- Improved Computational Efficiency: Although traditional full-wave electromagnetic simulation approaches the physical limit, each parameter iteration requires 3D mesh generation and matrix inversion, resulting in computation times of up to several hours. However, with the introduction of Gaussian Process Regression surrogate models, all algorithms shift the computational domain to a mathematical space where operations take only seconds, drastically reducing the total optimization time from “hours” to “seconds” and resolving the issue of excessively long design times in traditional methods.
- Improved Optimization Accuracy and Global Search Capability: A comparison of the Particle Swarm Optimization algorithm and the Genetic Algorithm revealed that while the Particle Swarm Optimization converges extremely rapidly in the early stages, it is prone to getting stuck in local minima due to a loss of population diversity when dealing with the complex nonlinear parameter space resulting from on-chip inductance in multilayer metal interconnects, leading to relatively large errors. In contrast, the Genetic Algorithm, with its unique roulette wheel selection, arithmetic crossover, and Gaussian mutation mechanisms, is able to effectively escape local optima, ultimately controlling the comprehensive error between the predicted inductance and the target value to around 2%; however, the algorithm’s convergence rate is relatively slow. In contrast, by combining the particle swarm optimization algorithm with the genetic algorithm—leveraging the fast convergence of Particle Swarm Optimization and the genetic algorithm’s ability to escape local optima—the comprehensive error between the predicted inductance and the target value was ultimately controlled to less than 1%, while maintaining a reasonable convergence rate.

In addition, we should also note that the mean relative errors shown in Table 2 reflect the errors of the GPR surrogate model in fitting *R_s_*, *L_s_*, and *C_ox_*. The optimization accuracy of less than 1% refers to the deviation between the inductance values and quality factors predicted by the surrogate model and their respective design targets.

### 3.3. Verification of Algorithm Optimization Results

**Figure 5:**
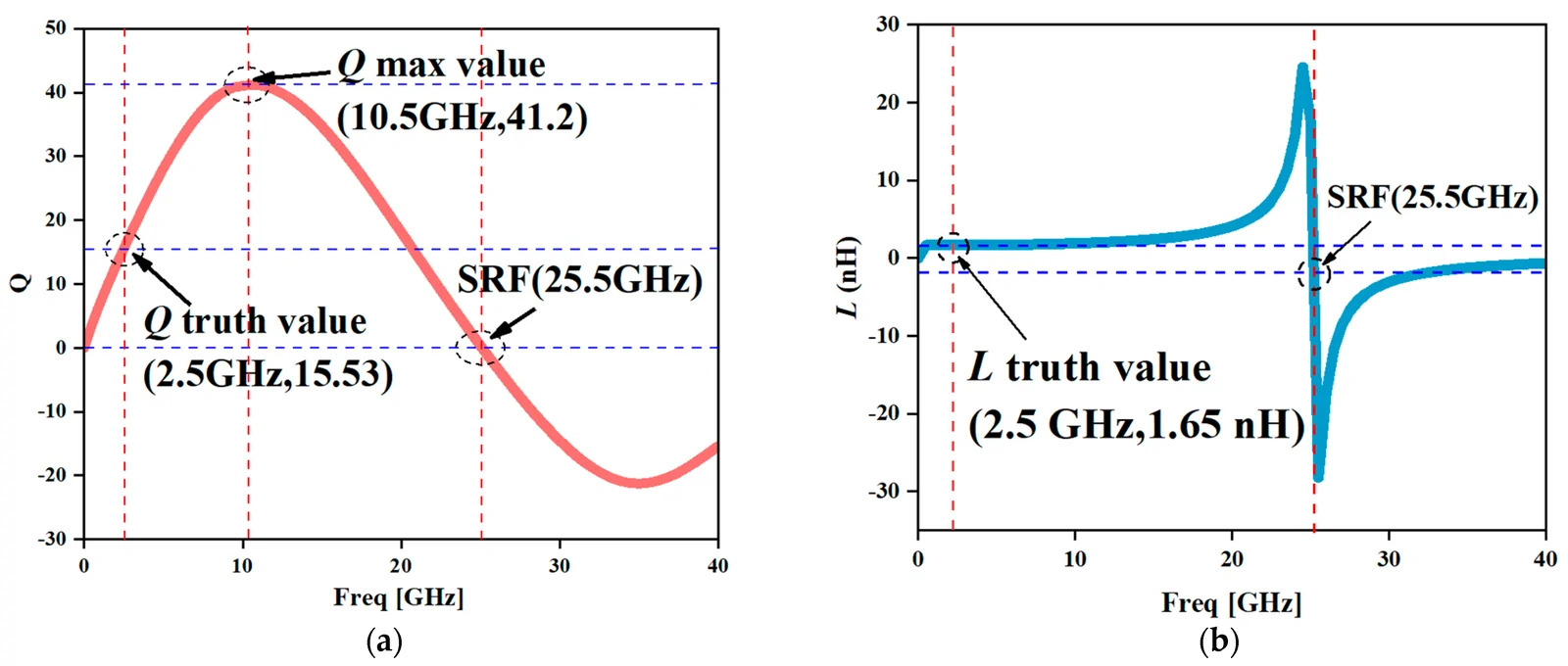
HFSS simulation results of the optimized on-chip inductor: (**a**) Quality factor versus frequency; (**b**) Inductance versus frequency.

To verify the electromagnetic performance of the optimized inductor, the final geometric model obtained through PSO-GA optimization was re-simulated using HFSS’s full-wave electromagnetic solver. At a frequency of 2.5 GHz, the GPR surrogate model predicted an inductance value of 1.587 nH and a quality factor of 15.45, while the corresponding HFSS simulation results were 1.650 nH and 15.53, respectively. Using the HFSS results as a reference, the relative deviation for inductance was 3.818%, and the relative deviation for the quality factor was 0.515%. The comparison results indicate that the surrogate model provides a relatively accurate prediction of the quality factor but exhibits some deviation in the prediction of inductance. The prediction results and the HFSS simulation results are shown in Table 4. The results of the HFSS simulation are shown in Figure 5.

In addition, we compared our results with those of recent research on machine learning-assisted on-chip inductor design; the comparison results are shown in Table 5.

### 3.4. Computational Cost Analysis

The computational cost of the framework proposed in this study consists primarily of three components: the time spent using automated scripts to call HFSS to generate the dataset, the time spent training the GPR surrogate model, and the time spent on algorithm optimization. The specific time spent is shown in Table 6.

Although generating the raw dataset and training the GPR surrogate model take a considerable amount of time, these are one-time efforts; once the dataset and the GPR surrogate model have been built, these two steps are no longer required for subsequent algorithm optimization.

## 4. Conclusions

This paper addresses the challenges in RFIC design—namely, the complex parameter space of on-chip passive inductor structures, the excessive time required for physical electromagnetic simulation, and the tendency of traditional optimization algorithms to get stuck in local optima—by designing and implementing a comprehensive, efficient framework for automated modeling and inverse optimization.

This study implemented low-level, deep communication with the Ansys HFSS full-wave electromagnetic simulator using Python 3.9 and the PyAEDT interface. It established an automated workflow for the modeling, port definition, solution calculation, and data analysis and extraction of highly flexible, parametric on-chip octagonal helical inductors, transforming the previously cumbersome manual modeling and frequency-sweep extraction process into a seamlessly operating automated workflow.

To address the issues of time-consuming and inefficient full-wave simulation calculations, this paper innovatively employs a physical component decoupling mechanism. Using the Gaussian Process Regression algorithm, it establishes a high-precision, physics-driven surrogate model that maps inductor geometric parameters (line width, line spacing, number of turns, inner diameter) to core high-frequency physical parameters (series inductance, series resistance, oxide layer capacitance). Experimental validation shows that the coefficient of determination (*R*^2^) for each component’s fit reached 0.970 or higher. This not only perfectly replicates the high-precision electromagnetic characteristics of electromagnetic simulations but also enables rapid performance prediction within seconds.

Finally, this paper successfully integrates the global rapid convergence characteristics of the particle swarm optimization algorithm with the spatial random perturbation mechanism of the genetic algorithm’s mutation operator, thereby proposing a PSO-GA hybrid intelligent optimization strategy. After setting constraints on the target quality factor and inductance value at a frequency of 2.5 GHz, the algorithm was able to effectively overcome the obstacle of local minima in the high-dimensional parameter space. The optimization results show that the relative error between the predicted inductance performance and the target value for the recommended geometric parameters is controlled within 0.9%, fully meeting the high-precision design standard of a deviation of less than 1%. At the same time, the entire inverse optimization search process took only about 4 s, achieving a breakthrough in efficiency compared to traditional electromagnetic simulation methods that involve exhaustive parameter sweeps (which typically take several hours or even longer).

In summary, the hybrid optimization framework for automated modeling and agent-based modeling proposed in this paper enables high-precision intelligent reverse design of on-chip inductor structures, significantly shortening the development cycle of passive components for RF front-end circuits. It holds significant engineering value and academic significance for enhancing the overall efficiency of automated RF integrated circuit design.

## Figures and Tables

**Table 1:** The value ranges of the geometric parameters for the on-chip inductors.

Parameters	Range
Trace width (*W*/µm)	6–12
Trace spacing (*S*/µm)	2–6
Number of turns (*N*)	2–5
Inner diameter (*D_in_*/µm)	60–100

**Table 2:** Prediction accuracy for gaussian process regression surrogate models.

Physical Components	Forecast Range	Coefficient of Determination	Average Relative Error
Series resistance	2.6–3.4 Ω	0.976	4.51%
Series inductance	1.4–1.9 nH	0.973	4.71%
Oxide layer capacitance	18–21 fF	0.982	6.64%

**Table 3:** Comparison table of the actual results of different optimization methods.

Optimization Methods	Minimum Error	Maximum Error	Average Error	Average Optimization Time
Particle swarm optimization	1.3%	5.9%	~2.2%	~3 s
Genetic algorithms	1.2%	3.5%	~1.7%	~7 s
Hybrid optimization algorithms	0.07%	1.9%	~0.7%	~4 s

**Table 4:** Comparison of predicted results with HFSS simulation results.

Parameter	GPR Prediction	HFSS Simulation	Error
Inductance	1.587 nH	1.650 nH	~3.82%
Quality factor	15.45	15.53	~0.52%

**Table 5:** Comparison of machine learning-assisted methods for on-chip inductor design.

Study	Modeling and Optimization Method	Prediction Accuracy	Optimization Time
[20]	Kriging surrogate modeling	Surrogate relative RMS error: 10.5% for L, 6.8% for *Q*	Model setup: 49.6 h
[21]	ANN + vector fitting + NSGA-II	The prediction results are similar to the actual results	Approximately 30 min after ANN training
[22]	GPR surrogate modeling + genetic algorithm	Error below 5%	No specific optimization times
This work	GPR surrogate modeling + PSO-GA Hybrid Algorithm	Optimization error: <1%. *Q* truth value error: ~0.5% L truth value error: ~3.8%	Generate the dataset: ~37 h Train model: ~7 min Algorithm to optimize: ~4 s

**Table 6:** The specific time spent on each stage.

The Stage	Time Cost
Generate the original dataset	~37 h
Training GPR surrogate Model	~7 min
Algorithmic optimization	~4 s

## Data Availability

The original contributions presented in this study are included in the article. Further inquiries can be directed to the corresponding authors.
